# Correlation between gene polymorphisms and perioperative analgesia in patients undergoing gynecological surgery

**DOI:** 10.3389/fgene.2025.1509042

**Published:** 2025-04-17

**Authors:** Meng Cai, Xiaofeng Lei, Lin Gan, Jing Li, Jin Yu

**Affiliations:** Department of Anesthesiology, Chongqing Health Center for Women and Children; Women and Children’s Hospital of Chongqing Medical University, Chongqing, China

**Keywords:** postoperative analgesia, single nucleotide polymorphism, gynecological laparoscopic operation, pharmacogenomics, personalized medicine

## Abstract

**Objectives:**

This study aims to identify specific single nucleotide polymorphism (SNP) correlated to perioperative analgesia in patients undergoing laparoscopic gynecological surgery.

**Methods:**

A total of 200 females meeting specific criteria underwent gynecological laparoscopic procedures under general anesthesia. Preoperative pain sensitivity was evaluated using Pain Sensitivity Questionnaire and Pain Catastrophizing Scale (PCS). Venous blood samples were collected for SNP analysis of nine genes. The study analyzed the correlation between SNPs and pre-operative pain assessment, analgesics usage, and the occurrence of related adverse effects.

**Results:**

Six out of nine identified loci showed polymorphisms. The PCS scores were higher in the mutation group (GG + GC) for *ADRA2A rs1800544* compared to the CC group (P < 0.05). No differences were observed in visual analog scale or Ramsay sedation scores between the mutation and wild-type groups for any of the SNPs (P < 0.05). Patients in the mutant group (AG + GG) for *OPRM1 rs1799971* had higher analgesic usage within 24 h compared to the wild-type group (P < 0.05). The consumption of intraoperative remifentanil was higher in the mutation group (GG + GC) of *ADRA2A rs1800544* than in the CC group. The Multifactorial Dimensionality Reduction analysis suggests that the optimal interaction model includes *OPRM1 rs179971* and *CYP450 3A4* * *1G rs2242480* together.

**Conclusion:**

Patients with GG and AG genotypes of *OPRM1 rs1799971* gene required more 24-h postoperative analgesics after gynecological surgery compared to those with AA genotype. A SNP-SNP interaction was observed between *OPRM1 rs179971* and CYP450 3A4 * 1G rs2242480.

**Clinical Trial Registration:** (www.chictr.org.cn, registration number: ChiCTR2200062425)

## 1 Introduction

Establishing safe and efficient postoperative pain relief is crucial for promoting a speedy recovery from surgery. However, the experience of pain and the effectiveness of pain medications can vary among patients undergoing gynecological surgery, influenced by factors such as age, physical health, and the severity of their condition. Additionally, genetic variability plays a significant role in these individual differences (Liu et al., 2020; Frangakis et al., 2023).

Sufentanil, a fentanyl analog, primarily acts on μ-opioid receptors and is commonly used as an intravenous, subarachnoid, and epidural anesthetic, as well as a postoperative analgesic due to its strong efficacy and high lipid solubility. The μ-opioid receptor, targeted by sufentanil, is encoded by the OPRM1 gene (OPRM1) (Oertel et al., 2009). Among the variants of the OPRM1 gene, the A118G variant is the most prevalent single nucleotide polymorphism (SNP) (Lotsch et al., 2002). The *OPRM1 (rs1799971)* variant results in the substitution of asparagine with aspartate at position 40, leading to elevated pain scores and increased morphine usage (Yao et al., 2015). Within the CYP3 enzyme family, CYP3A4 is a crucial hepatic microsomal enzyme responsible for metabolizing 45%–60% of therapeutic medications including opiates and benzodiazepines (Burk and Wojnowski, 2004; Shu et al., 2024). Parturients with CT heterozygotes and TT homozygotes of CYP450 3A4*1G (rs2242480) required a lower dosage of sufentanil during epidural labor analgesia (Chi et al., 2015).

Dexmedetomidine, a selective α2 adrenergic receptor (α2-AR) agonist commonly used for postoperative pain relief, has been found to exhibit varying sedative effects based on genetic polymorphisms associated with ADRA2A (Zhu et al., 2019). Individuals with the G allele of ADRA2A (C1291G) typically require higher levels of sedation when administered dexmedetomidine compared to those with the C allele (Yagar et al., 2011). Specific variants of ADRA2A, such as *rs11195418*, *rs1800544*, *rs553668*, and *rs10885122*, have shown significant correlations with different sympathetic responses (Weerink et al., 2017).

Exploring the impact of genetic variants and pain-related SNP loci on the efficacy of opioid pain relief could lead to personalized postoperative analgesia management strategies based on individual genetic profiles. This approach has the potential to improve pain control, and reduce side effects such as nausea, vomiting and respiratory depression. Based on SNPs exhibiting documented variations in prior pharmacogenetic studies, we prioritized 9 loci with pharmacogenomic relevance to the therapeutics employed in this investigation, encompassing genetic variants associated with opioid receptors, metabolism, pain sensitivity and dexmedetomidine receptor polymorphisms. We analyzed SNPs of OPRM1 rs1799971, ADRA2A rs201376588, ADRA2A rs1800544, ADRA2A rs775887911, ADRA2A rs1800035, CYP450 3A4*1G rs2242480, COMT Val158met rs4680, SLC632 rs40615, and *SLC6A2 rs36006* to investigate their impact on postoperative analgesia in patients undergoing laparoscopic surgery. This study aims to identify specific genetic markers associated with postoperative pain management, laying the foundation for personalized analgesia administration.

## 2 Materials and methods

### 2.1 Study oversight

This study was approved by the Research Ethics Committee of Chongqing Health Center for Women and Children (Approval Number: 2022–006) and registered at the Chinese Clinical Trial Registry (ChiCTR) (www.chictr.org) with registration ID ChiCTR2200062425. The trial was conducted in accordance with the Declaration of Helsinki and written informed consent was obtained from all participants.

### 2.2 Participants

Two hundred Chinese female patients aged between 18 and 60 years, classified according to the American Society of Anesthesiologists (ASA) physical status I–III underwent gynecological laparoscopic surgeries under general anesthesia from 10 August 2022, to 30 December 2023, were included in this study. The inclusion criteria were as follows: 1) Planned gynecological laparoscopic surgery under general anesthesia with the need for postoperative patient-controlled intravenous analgesia (PCA). 2) Patients with proficient communication skills, capable of correctly using PCA pump, and willing to collaborate in assessing pain intensity. 3) Patients with no history of allergic reactions to narcotic drugs. 4) Patients able to comprehend the information in the informed consent form, make independent judgments, express willingness to participate, and sign the informed consent form. Exclusion criteria included: 1) Mental illness or cognitive impairment. 2) Allergy to known analgesic and sedative drugs. 3) History of chronic pain and prolonged use of analgesic and sedative drugs. 4) Patients with peripheral neuropathy symptoms or abnormal liver or kidney function. Removal criteria included surgical procedures lasting more than 3 h and severe complications such as significant bleeding or cardiac arrest during surgery.

### 2.3 Anesthesia and analgesia methods

In the operating room, patients underwent standard monitoring, including electrocardiography, noninvasive arterial pressure measurements and pulse oximetry. Oxygen was consistently administered at a rate of 5 L/min before anesthesia induction. Anesthesia induction was performed using simultaneous target-controlled infusion (TCI) of remifentanil at a concentration of 5 ng/mL and propofol at 5 μg/mL. Intravenous injections of rocuronium bromide at 0.6 mg/kg and sufentanil at 0.25 μg/kg were also given. Anesthesia maintenance included propofol TCI at 3.5 μg/mL and remifentanil at 6 ng/mL, with additional doses of sufentanil and rocuronium administered as needed. Patients were provided with self-controlled intravenous analgesia through a PCA pump after surgery (PCA-100C, Zhejiang Chen He Medical Equipment Co., Ltd., China). The analgesic solution consisted of dexmedetomidine (100 µg) and sufentanil (75 µg) diluted in 100 mL of normal saline. The PCA pump settings included a lockout interval of 15 min, a bolus dose of 0.5mL, and an infusion rate of 2 mL/h.

### 2.4 Data collection

Patient details were collected before surgery, and pain sensitivity was evaluated. Venous blood samples were collected for cryopreservation to analyze specific SNPs. The Pain Sensitivity Questionnaire (PSQ) and the Pain Catastrophizing Scale (PCS) were administered to all patients preoperatively. Total, moderate, and mild PSQ scores, as well as PCS scores, were calculated. Higher scores indicate increased sensitivity to pain stimuli.

Postoperative pain intensity was assessed using the visual analog scale (VAS) ranging from 0 to 10. Sedation level was evaluated using a five-point sedation scale: 1 for fully awake, 2 for mild sedation, 3 for lethargy, 4 for easily awakened from sleep, and 5 for deep sleep with difficulty awakening. The VAS and Ramsay sedation scores were recorded at 2-hour intervals up to 24 h post-surgery.

Postoperative 24-hour analgesic consumption, number of patient-controlled analgesia compressions within 24 h, and the time when analgesics in pump is completely depleted were recorded. Side effects including hypotension, bradycardia, nausea, vomiting, dizziness, urinary retention and shivering were also documented. Maternal hypotension was defined as a 20% decrease in noninvasive mean blood pressure from baseline. Bradycardia was identified as a heart rate below 60 beats per minute, and urinary retention was defined as the inability to urinate despite a full bladder.

### 2.5 Genotyping and single-nucleotide polymorphisms

Blood samples (2 mL) were collected and preserved in an EDTA anticoagulant tube before the surgical procedure. The samples were stored in a −80°C freezer for unified testing after gradient cooling. The extracted DNA was confirmed to meet the required criteria through a standardized extraction process. Genotype analysis of all the SNPs was conducted using MassARRAY SNP typing technology. Table 1 shows the PCR primers and UEP primer for the 9 SNP loci. The Spectra CHIP instrument was used for analysis with a MALDI-TOF mass spectrometer, and TYPER 4.0 software was used to obtain raw data and genotype maps from the detection results. Agena Bioscience, Inc.'s 384-well Spectro CHIP^®^ Bioarray chip, MassARRAY Nano Inspector Point Prototype, and MassARRAY Analyzer 4.0 mass spectrometer were utilized.

**TABLE 1:** Primers for each SNP locus.

SNP ID	2nd-PCRP Reverse primer sequence 5'->3′	1st-PCRP Forward primer sequence 5'->3′	UEP_SEQ Single base extension primer sequence	Detection rate
*ADRA2A rs201376588*	ACG​TTG​GAT​GAG​CAG​GCC​CAG​GCC​AGC​G	ACG​TTG​GAT​GGC​GTC​CGG​CTG​CAG​GGA​G	CCCGCGTTCATGTTC	100%
*OPRM1 rs1799971*	ACG​TTG​GAT​GAG​GGC​ACA​GGC​TGT​CTC​TC	ACG​TTG​GAT​GGT​TCC​TGG​GTC​AAC​TTG​TCC	ATGGGTCGGACAGGT	100%
*ADRA2A rs1800544*	ACG​TTG​GAT​GCC​TGC​TGG​GAG​TTG​GCC​AT	ACG​TTG​GAT​GTT​CTC​CCA​AGA​TCC​AGC​TTC	GTTGGCCATGCAGCTC	100%
CYP450 3A4*1G rs2242480	ACG​TTG​GAT​GTG​CTA​AGG​TTT​CAC​CTC​CTC	ACG​TTG​GAT​GGC​AGG​AGG​AAA​TTG​ATG​CAG	CCCTCCTTCTCCATGTA	100%
*COMT Val158met rs4680*	ACG​TTG​GAT​GTT​TTC​CAG​GTC​TGA​CAA​CGG	ACG​TTG​GAT​GAC​CCA​GCG​GAT​GGT​GGA​TTT	GCACACCTTGTCCTTCA	99%
*ADRA2A rs775887911*	ACG​TTG​GAT​GGC​GTC​CGG​CTG​CAG​GGA​G	ACG​TTG​GAT​GAG​CAG​GCC​CAG​GCC​AGC​G	GCTGCAGGGAGCCCATG	99%
*ADRA2A rs1800035*	ACG​TTG​GAT​GTC​CGG​ACG​CCG​TCG​CCG​C	ACG​TTG​GAT​GTT​CGG​CCT​CTG​CGC​CCC​C	CCCCGAGCGCAGGCCCAA	99%
*SLC632 rs40615*	ACG​TTG​GAT​GTT​TGA​TCA​GAT​ACC​CCT​CCC	ACG​TTG​GAT​GAA​CCA​CCC​CAC​TAA​ACT​CAG	CCT​CCC​AAA​AAA​AAA​AAA​AAC	99%
*SLC6A2 rs36006*	ACG​TTG​GAT​GGT​CAT​GCA​GAT​TTC​TGT​GTC	ACG​TTG​GAT​GGA​TAC​ACA​GCC​CAG​GAA​ACA	GGA​TTT​TAA​AAG​TTG​TTC​TCT​T	98%

PCR, Polymerase chain reaction. UEP, unique event polymorphism.

The SNP genotyping for all samples in this study was conducted by professional genetic testing technicians at Beijing Liuhe BGI Co., Ltd. Quality control measures included checking the OD values of all DNA samples using the NanoDrop2000 instrument, performing 1.25% agarose gel electrophoresis testing, and assessing the quality to ensure compliance with MassARRAY SNP genotyping DNA quality requirements. The quality control standards were as follows: DNA concentration greater than 20 ng/μL, OD260/280 value between 2.2 and 1.6, OD260/230 value greater than 0.6, no absorption peak at OD230 nm, and intact gDNA without severe degradation in the DNA electrophoresis result. Primer design was conducted using Agena’s Assay Designer 4.0 software for evaluating multi-SNP locus primer design. Design parameters were adjusted based on the information for different loci to meet optimization standards. SNP genotyping results were obtained by calculating the peak area size of DNA products for all SNP loci, and the final genotyping accuracy was clearly assessed through a cluster quality control chart.

### 2.6 SNP-SNP interaction analysis

SNP-SNP interaction analysis was conducted using Multifactorial Dimensionality Reduction (MDR) (Fu et al., 2020; Ritchie et al., 2001). The Fruchterman-Reingold diagram represented the interaction intensity between different SNP sites as ‘n %'. A higher value indicated a stronger interaction between the two SNP sites. The proximity of SNP sites in the tree diagram reflected the strength of their interaction with closer distances indicating stronger interactions and greater distances indicating weaker interactions. A postoperative VAS score of 3 or higher was considered a positive outcome indicator.

### 2.7 Statistical analysis

Statistical analysis was performed using SPSS version 21.0 for Windows (SPSS, Inc., Chicago, IL, USA). Genotype and allele frequencies for each locus were calculated, using χ2 to verify whether the distribution of each genotype conformed to the Hardy–Weinberg equilibrium. Mean and standard deviation were calculated for continuous data, while count values were used for categorical data. Normality of continuous data was assessed using the Kolmogorov-Smirnov test. Independent-sample t-tests were used for comparing continuous data between groups, and the Kruskal–Wallis test was used for non-parametric data. Categorical data were compared using the chi-square test or Fisher’s exact test. A p-value <0.05 was considered statistically significant.

## 3 Results

### 3.1 Patient demographics and gene polymorphism characteristics

**FIGURE 1:**
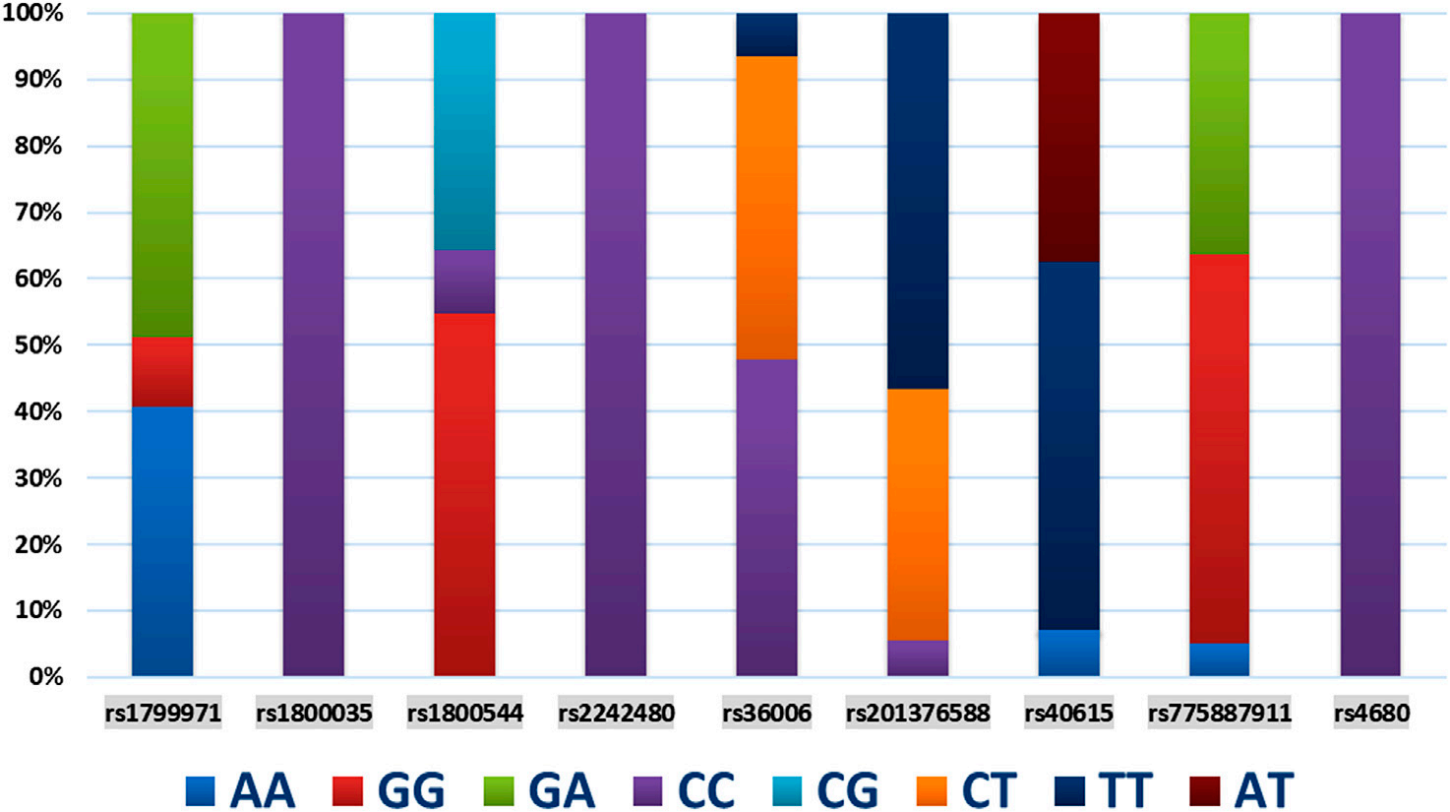
The distribution and composition mapping of gene loci.

The demographic characteristics, including age, weight, height, body mass index, and operation time, of each SNP of genes are presented in Table 2. Among the 9 SNPs identified, 6 loci showed polymorphisms: OPRM1 rs1799971, CYP450 3A4 * 1G rs2242480, SLC6A2 rs36006, SLC632 rs40615, COMT Val158met rs4680, and *ADRA2A rs1800544*. The distribution of loci for each SNP of genes is displayed in Table 3 and Figure 1. The frequency of the *OPRM1 rs1799971* G allele was 35%, while the A allele frequency was 65%. The frequency of the *ADRA2A rs1800544* G allele was 73%, and the C allele frequency was 27% (Table 3). No significant difference was observed between the wild-type and mutant groups (*P* > 0.05).

**TABLE 2:** Patient demographic parameters for each SNP of genes.

SNP	Age (y)	Weight (kg)	Height (cm)	Body mass index (kg/m^2^)	Operation time (min)
*OPRM A118G rs1799971*					
AA (n = 81)	37.91 ± 9.59	56.67 ± 8.19	158.94 ± 5.42	22.51 ± 3.19	97.79 ± 40.98
AG + GG (n = 118)	40.36 ± 9.74	57.94 ± 8.36	158.85 ± 4.42	23.07 ± 3.40	113.19 ± 49.52
*P* value	0.08	0.29	0.89	0.23	0.18
*ADRA2A C1291G rs1800544*					
CC (n = 19)	39.44 ± 9.69	57.38 ± 8.29	158.94 ± 4.91	22.81 ± 3.33	104.85 ± 45.33
CG + GG (n = 180)	38.63 ± 10.35	57.89 ± 8.60	158.42 ± 4.23	23.13 ± 3.27	126.53 ± 56.16
*P* value	0.73	0.79	0.65	0.69	0.15
CYP450 3A4*1G rs2242480					
CC (n = 95)	38.42 ± 10.01	57.94 ± 8.85	158.72 ± 4.79	23.15 ± 3.54	107.07 ± 48.14
CT + TT (n = 104)	40.23 ± 9.44	56.96 ± 7.78	159.06 ± 4.91	22.57 ± 3.10	106.78 ± 45.67
*P* value	0.19	0.41	0.63	0.22	0.97
*COMT Val158met rs4680*					
GG (n = 114)	39.82 ± 9.62	57.63 ± 8.10	158.49 ± 4.42	23.08 ± 3.46	107.49 ± 49.76
GA + AA (n = 81)	38.47 ± 9.81	57.10 ± 8.54	159.27 ± 5.35	22.58 ± 3.14	107.26 ± 43.04
*P value*	0.34	0.66	0.27	0.30	0.97
*SLC6A2 rs36006*					
TT (n = 112)	39.56 ± 9.72	57.74 ± 8.35	158.93 ± 5.07	22.97 ± 3.37	17.79 ± 43.13
CT + CC (n = 86)	39.26 ± 9.77	57.06 ± 8.30	158.83 ± 4.59	22.72 ± 3.30	105.86 ± 51.56
*P* value	0.83	0.57	0.89	0.61	0.78
*SLC6A2 rs40615*					
TT (n = 109)	39.83 ± 9.67	57.63 ± 8.43	158.91 ± 5.16	22.93 ± 3.42	107.46 ± 43.47
AT + AA (n = 88)	39.07 ± 9.80	57.29 ± 8.24	158.81 ± 4.49	22.80 ± 3.24	106.75 ± 51.10
*P* value	0.59	0.78	0.89	0.79	0.92

The data are expressed as the mean ± SD, or n (%).

**TABLE 3:** The distribution of loci for each SNP of genes.

SNP	Genotype	n (%)	Allele	n (%)	H-W *P* Value	Genotyping rate
*OPRM A118G rs1799971*	AA	81 (40.70)	A	65.08%	0.773	99.5%
	AG	97 (48.74)	G	34.92%		
	GG	21 (10.55)				
*ADRA2A C1291G rs1800544*	CC	19 (9.50)	C	27.39%	0.615	99.5%
	CG	71 (35.68)	G	72.61%		
	GG	109 (54.77)				
CYP450 3A4*1G rs2242480	CC	95 (47.74)	C	70.60%	0.612	99.5%
	CT	91 (45.73)	T	29.40%		
	TT	13 (6.53)				
*COMT Val158met rs4680*	AA	10 (5.13)	A	23.33%	0.972	97.5%
	GA	71 (36.41)	G	76.67%		
	GG	114 (58.46)				
*SLC6A2 rs36006*	CC	11 (5.56)	C	24.49%	0.963	99%
	CT	75 (37.88)	T	75.51%		
	TT	112 (56.57)				
*SLC6A2 rs40615*	TT	109 (55.33)	T	74.11%	0.966	98.5%
	AT	74 (37.56)	A	25.89%		
	AA	14 (7.11)				
*ADRA2A rs1800035*	CC	199 (100)	C	398 (100%)	—	99.5%
*ADRA2A rs201376588*	CC	197 (100)	C	394 (100%)	—	98.5%
*ADRA2A rs775887911*	CC	197 (100)	C	394 (100%)	—	98.5%

H-W, Hardy‒Weinberg equilibrium.

### 3.2 Pain score

**FIGURE 2:**
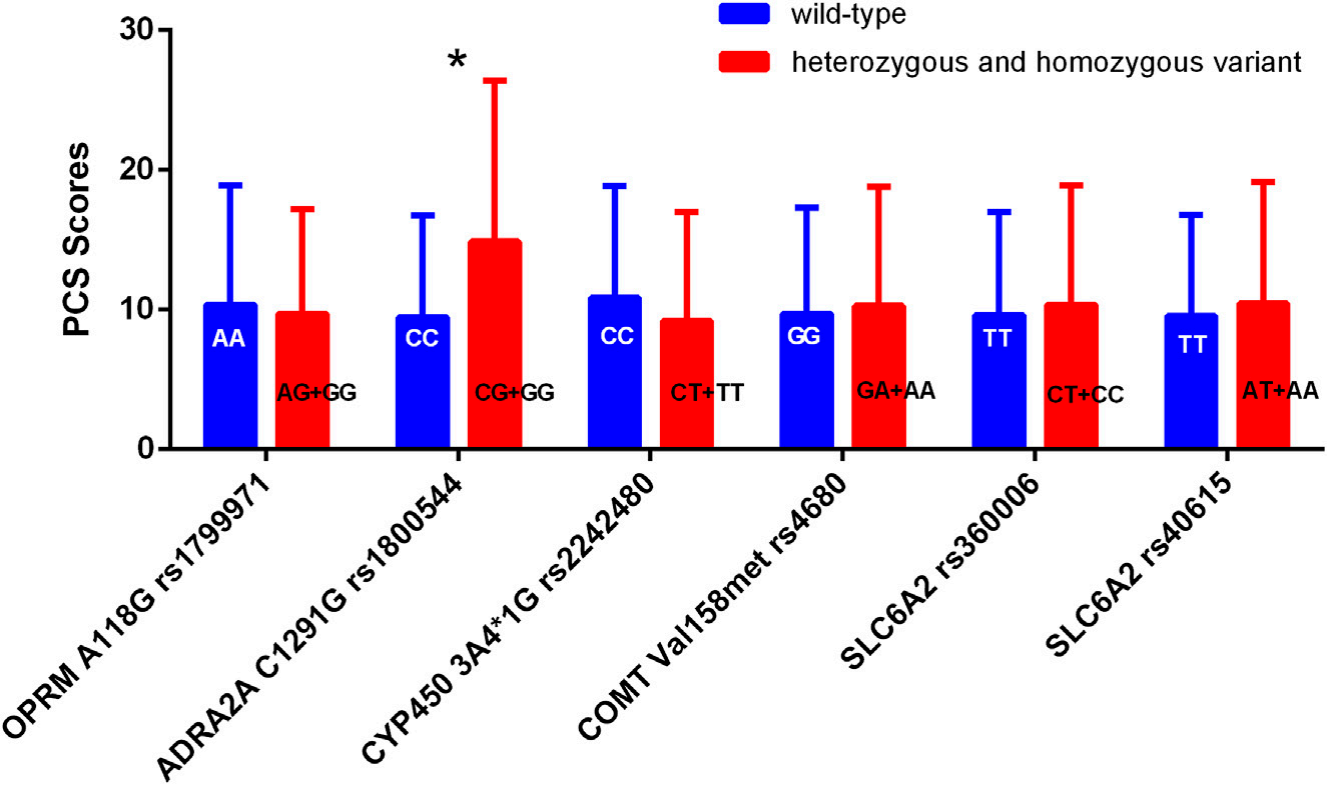
Comparison of PCS scores among patients with different genotypes. *P < 0.05 between wild-type and heterozygous or homozygous variant.

No statistically significant differences were found in PCS or PSQ scores between the wild-type and mutant groups, except for *ADRA2A rs1800544* (*P* > 0.05). The PCS scores of individuals in the mutation group (GG + GC) for *ADRA2A rs1800544* were significantly higher than those of the CC group (*P* < 0.05) (Table 4; Figure 2).

**TABLE 4:** Comparison of PCS and PSQ scores between patients with different genotypes.

SNP	PCS scores	PSQ total scores	PSQ minor scores	PSQ moderate scores
*OPRM A118G rs1799971*				
AA (n = 81)	10.37 ± 8.54	4.24 ± 1.03	3.44 ± 1.09	4.62 ± 1.06
AG + GG (n = 118)	9.71 ± 7.48	4.07 ± 1.03	3.42 ± 1.03	4.42 ± 1.04
*P* value	0.56	0.27	0.89	0.17
*ADRA2A C1291G rs1800544*				
CC (n = 19)	9.46 ± 7.28	4.13 ± 1.03	3.42 ± 1.03	4.48 ± 1.05
CG + GG (n = 180)	14.89 ± 11.51	4.27 ± 1.08	3.56 ± 1.25	4.69 ± 1.06
*P* value	0.01*	0.57	0.58	0.40
CYP450 3A4*1G rs2242480				
CC (n = 95)	10.84 ± 8.00	4.23 ± 1.01	3.53 ± 1.07	4.57 ± 1.04
CT + TT (n = 104)	9.19 ± 7.79	4.07 ± 1.05	3.34 ± 1.04	4.44 ± 1.07
*P* value	0.14	0.27	0.20	0.38
*COMT Val158met rs4680*				
GG (n = 114)	9.72 ± 7.58	4.13 ± 1.08	3.46 ± 1.13	4.50 ± 1.10
GA + AA (n = 81)	10.28 ± 8.53	4.16 ± 0.99	3.39 ± 0.97	4.51 ± 1.02
*P* value	0.63	0.85	0.62	0.94
*SLC6A2 rs36006*				
TT (n = 112)	9.60 ± 7.39	4.05 ± 1.03	3.36 ± 1.00	4.42 ± 1.03
CT + CC (n = 86)	10.35 ± 8.54	4.26 ± 1.04	3.52 ± 1.12	4.61 ± 1.09
*P* value	0.51	0.16	0.28	0.21
*SLC6A2 rs40615*				
TT (n = 109)	9.57 ± 7.21	4.09 ± 1.01	3.37 ± 1.01	4.45 ± 1.04
AT + AA (n = 88)	10.44 ± 8.70	4.19 ± 1.07	3.49 ± 1.11	4.56 ± 1.08
*P* value	0.44	0.50	0.43	0.47

The data are presented as the means ± SDs; *P < 0.05.

There were no statistically significant differences in VAS score or Ramsay score between the mutation group and the wild-type group at any of the SNPs across the different time points (*P* > 0.05). (Tables 5, 6).

**TABLE 5:** Comparison of VAS scores between different genotypes.

SNP	VAS score				
	T1	T2	T3	T4	T5
*OPRM A118G rs1799971*					
AA (n = 81)	2.95 ± 1.85	2.22 ± 1.60	1.91 ± 1.68	1.47 ± 1.37	1.20 ± 1.15
AG + GG (n = 118)	2.90 ± 1.98	2.31 ± 1.56	1.82 ± 1.55	1.39 ± 1.37	1.14 ± 1.22
*P* value	0.85	0.69	0.69	0.69	0.72
*ADRA2A C1291G rs1800544*					
CC (n = 19)	2.91 ± 1.91	2.29 ± 1.59	1.89 ± 1.63	1.41 ± 1.36	1.15 ± 1.18
CG + GG (n = 180)	3.05 ± 2.12	2.11 ± 1.44	1.53 ± 1.30	1.53 ± 1.38	1.26 ± 1.28
*P* value	0.75	0.62	0.34	0.72	0.69
CYP450 3A4*1G rs2242480					
CC (n = 95)	2.84 ± 1.65	2.34 ± 1.40	1.96 ± 1.59	1.52 ± 1.39	1.25 ± 1.18
CT + TT (n = 104)	2.99 ± 2.16	2.22 ± 1.74	1.77 ± 1.62	1.34 ± 1.34	1.08 ± 1.21
*P* value	0.59	0.61	0.41	0.36	0.30
*COMT Val158met rs4680*					
GG (n = 114)	2.89 ± 1.57	2.28 ± 1.35	1.92 ± 1.57	1.53 ± 1.40	1.29 ± 1.20
GA + AA (n = 81)	3.00 ± 2.32	2.30 ± 1.85	1.79 ± 1.67	1.30 ± 1.34	1.01 ± 1.18
*P value*	0.68	0.95	0.58	0.25	0.11
*SLC6A2 rs36006*					
TT (n = 112)	3.09 ± 1.98	2.39 ± 1.61	1.93 ± 1.62	1.54 ± 1.43	1.27 ± 1.24
CT + CC (n = 86)	2.70 ± 1.85	2.15 ± 1.53	1.79 ± 1.59	1.29 ± 1.27	1.03 ± 1.12
*P* value	1.58	2.87	0.55	0.21	0.18
*SLC6A2 rs40615*					
TT (n = 109)	3.07 ± 1.89	2.42 ± 1.63	2.03 ± 1.71	1.61 ± 1.47	1.28 ± 1.25
AT + AA (n = 88)	2.65 ± 1.83	2.10 ± 1.49	1.69 ± 1.45	1.23 ± 1.21	1.03 ± 1.11
*P* value	0.11	0.16	0.15	0.05	0.14

T1: 2 h after surgery, T2: 4 h after surgery, T3: 6 h after surgery, T4: 12 h after surgery, T5: 24 h after surgery.

**TABLE 6:** Comparison of Ramsay scores between different genotypes.

SNP	Ramsay score				
	T1	T2	T3	T4	T5
*OPRM A118G rs1799971*					
AA (n = 81)	2.38 ± 0.48	2.01 ± 0.11	2.00 ± 0.00	2.00 ± 0.00	2.00 ± 0.00
AG + GG (n = 118)	2.33 ± 0.49	2.04 ± 0.24	2.01 ± 0.09	2.01 ± 0.09	2.01 ± 0.09
*P* value	0.46	0.29	0.40	0.40	0.40
*ADRA2A C1291G rs1800544*					
CC (n = 19)	2.36 ± 0.49	2.03 ± 0.20	2.01 ± 0.07	2.01 ± 0.07	2.01 ± 0.07
CG + GG (n = 180)	2.26 ± 0.45	2.00 ± 0.00	2.00 ± 0.00	2.00 ± 0.00	2.00 ± 0.00
*P* value	0.48	0.74	0.74	0.74	0.74
CYP450 3A4*1G rs2242480					
CC (n = 95)	2.38 ± 0.51	2.02 ± 0.21	2.01 ± 0.10	2.01 ± 0.10	2.01 ± 0.10
CT + TT (n = 104)	2.33 ± 0.47	2.04 ± 0.19	2.00 ± 0.00	2.00 ± 0.00	2.00 ± 0.00
*P* value	0.46	0.54	0.30	0.30	0.30
*COMT Val158met rs4680*					
GG (n = 114)	2.36 ± 0.48	2.04 ± 0.21	2.00 ± 0.00	2.00 ± 0.00	2.00 ± 0.00
GA + AA (n = 81)	2.33 ± 0.50	2.01 ± 0.19	2.01 ± 0.11	2.01 ± 0.11	2.01 ± 0.11
*P value*	0.71	0.28	0.24	0.24	0.24
*SLC6A2 rs36006*					
TT (n = 112)	2.29 ± 0.48	2.01 ± 0.16	2.01 ± 0.09	2.01 ± 0.09	2.01 ± 0.09
CT + CC (n = 86)	2.43 ± 0.50	2.06 ± 0.23	2.00 ± 0.00	2.00 ± 0.00	2.00 ± 0.00
*P* value	0.05	0.09	0.38	0.38	0.38
*SLC6A2 rs40615*					
TT (n = 109)	2.31 ± 0.49	2.01 ± 0.17	2.01 ± 0.09	2.01 ± 0.09	2.01 ± 0.09
AT + AA (n = 88)	2.41 ± 0.49	2.06 ± 0.23	2.00 ± 0.00	2.00 ± 0.00	2.00 ± 0.00
*P* value	0.17	0.09	0.37	0.37	0.37

T1, 2 h after surgery, T2, 4 h after surgery, T3, 6 h after surgery, T4, 12 h after surgery, T5, 24 h after surgery.

### 3.3 Analgesics consumption

**FIGURE 3:**
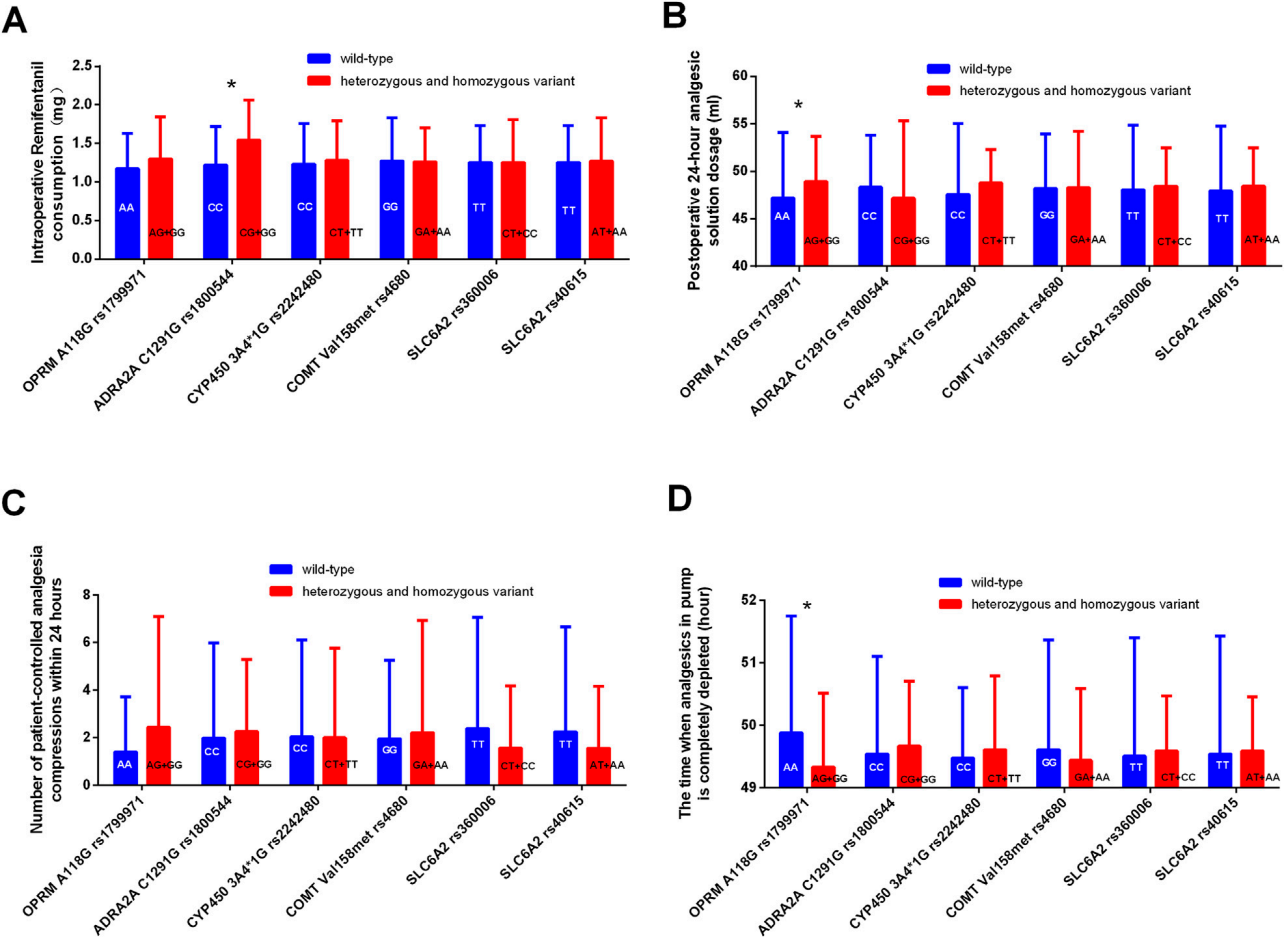
Comparison of analgesic consumption related indicators among different genotypes. **(A)** Comparison of intraoperative Remifentanil consumption between different genotypes. **(B)** Comparison of postoperative 24-hour analgesic solution dosage between different genotypes. **(C)** Comparison of patient-controlled analgesia compressions within 24 h between different genotypes. **(D)** Comparison of the time when analgesics in pump is completely depleted between different genotypes. *P < 0.05 between wild-type and heterozygous or homozygous variant.

The consumption of intraoperative remifentanil was higher in the mutation group (GG + GC) of *ADRA2A rs1800544* genotype compared to the CC group (Figure 3A). Compared to those in the wild-type *OPRM1 rs1799971* group, patients in the mutant group (AG + GG) showed increased analgesic solution consumption within 24 h (P < 0.05) (Figure 3B). No statistically significant differences were observed in analgesic usage between the mutation group and the wild-type group at other SNPs (Table 7; Figure 3).

**TABLE 7:** Comparison of analgesic consumption between different genotypes.

SNP	Intraoperative remifentanil consumption (mg)	Postoperative 24-h analgesic solution dosage (mL)	Number of patient-controlled analgesia compressions within 24 h	The time when analgesics in pump is completely depleted (hour)
*OPRM A118G rs1799971*				
AA (n = 81)	1.17 ± 0.46	47.17 ± 6.89	1.40 ± 2.31	49.88 ± 1.87
AG + GG (n = 118)	1.30 ± 0.54	48.91 ± 4.75	2.43 ± 4.67	49.33 ± 1.18
*P* value	0.07	0.03*	0.07	0.01*
*ADRA2A C1291G rs1800544*				
CC (n = 19)	1.22 ± 0.50	48.31 ± 5.48	1.98 ± 4.00	49.54 ± 1.56
CG + GG (n = 180)	1.54 ± 0.52	47.16 ± 8.14	2.26 ± 3.03	49.67 ± 1.04
*P* value	0.01*	0.41	0.71	0.64
CYP450 3A4*1G rs2242480				
CC (n = 95)	1.23 ± 0.53	47.55 ± 7.47	2.03 ± 4.09	49.48 ± 1.12
CT + TT (n = 104)	1.28 ± 0.51	48.80 ± 3.50	1.99 ± 3.77	49.61 ± 1.80
*P* value	0.46	0.13	0.94	0.54
*COMT Val158met rs4680*				
GG (n = 114)	1.27 ± 0.56	48.18 ± 5.76	1.95 ± 3.30	49.61 ± 1.76
GA + AA (n = 81)	1.26 ± 0.44	48.25 ± 5.97	2.20 ± 4.73	49.44 ± 1.15
*P value*	0.92	0.93	0.67	0.43
*SLC6A2 rs36006*				
TT (n = 112)	1.25 ± 0.48	48.03 ± 6.83	2.38 ± 4.67	49.51 ± 1.89
CT + CC (n = 86)	1.25 ± 0.56	48.43 ± 4.06	1.56 ± 2.62	49.59 ± 0.88
*P* value	0.99	0.63	0.15	0.71
*SLC6A2 rs40615*				
TT (n = 109)	1.25 ± 0.48	47.92 ± 6.85	2.24 ± 4.41	49.54 ± 1.89
AT + AA (n = 88)	1.27 ± 0.56	48.44 ± 4.02	1.55 ± 2.61	49.59 ± 0.87
*P* value	0.77	0.53	0.19	0.80

The data are presented as the means ± SDs; *P < 0.05.

### 3.4 Adverse reactions

No statistically significant differences were found in adverse reactions, including hypotension, bradycardia, nausea and vomiting, dizziness, urinary retention, or shivering, except for *ADRA2A rs1800544* (P > 0.05). Although a lower incidence of hypotension was observed in the mutation group (GG + GC) for *ADRA2A rs1800544* compared to the CC group, the difference was not statistically significant (P > 0.05) (Table 8).

**TABLE 8:** Comparison of adverse reactions between different genotypes.

SNP	Hypotension (%)	Bradycardia n (%)	Nausea n (%)	Vomiting n (%)	Dizziness n (%)	Urinary retention n (%)	Shiver n (%)
*OPRMA118G rs1799971*							
AA (n = 81)	2 (2.5%)	3 (3.7%)	13 (16.0%)	8 (9.8%)	1 (1.2%)	0 (0)	3 (3.7%)
AG + GG (n = 118)	2 (1.7%)	2 (1.7%)	17 (14.4%)	11 (9.3%)	4 (3.4%)	2 (1.7%)	8 (6.8%)
*P* value	1.00	0.67	0.75	0.89	0.62	0.65	0.53
*ADRA2A C1291G rs1800544*							
CC (n = 19)	2 (10.5%)	0 (0)	3 (15.8%)	1 (5.3%)	0 (0)	0 (0)	2 (10.5%)
CG + GG (n = 180)	2 (1.1%)	5 (2.8%)	28 (15.6%)	18 (10%)	5 (2.8%)	2 (1.1%)	9 (5%)
*P* value	0.06	1.00	1.00	0.79	1.00	1.00	0.63
CYP450 3A4*1G rs2242480							
CC(n = 95)	3 (3.2%)	2 (2.1%)	14 (14.7%)	9 (9.5%)	3 (3.2%)	1 (1.1%)	2 (2.1%)
CT + TT (n = 104)	1 (1.0%)	3 (2.9%)	17 (16.3%)	10 (9.6%)	2 (1.9%)	1 (1.0%)	9 (8.7%)
*P* value	0.55	1.00	0.76	0.97	0.92	0.55	0.09
*COMT Val158met rs4680*							
GG (n = 114)	2 (1.8%)	2 (1.8%)	19 (16.7%)	12 (10.5%)	2 (1.8%)	1 (0.9%)	9 (7.9%)
GA + AA (n = 81)	2 (2.5%)	3 (3.7%)	11 (13.6%)	7 (8.7%)	3 (3.7%)	1 (1.2%)	2 (2.5%)
*P* value	1.00	0.69	0.56	0.66	0.69	1.00	0.19
*SLC6A2 rs36006*							
TT (n = 112)	2 (1.8%)	4 (3.6%)	16 (14.3%)	10 (8.9%)	2 (1.8%)	2 (1.8%)	7 (6.3%)
CT + CC(n = 86)	2 (2.3%)	1 (1.2%)	15 (17.4%)	9 (10.5%)	3 (3.5%)	0 (0)	4 (4.7%)
*P* value	1.00	0.54	0.54	0.72	0.76	0.59	0.62
*SLC6A2 rs40615*							
TT (n = 109)	2 (1.8%)	4 (3.7%)	17 (15.6%)	10 (9.2%)	2 (1.8%)	1 (0.9%)	6 (5.5%)
AT + AA (n = 88)	2 (2.3%)	1 (1.1%)	14 (15.9%)	9 (10.2%)	3 (3.4%)	0 (0)	5 (5.7%)
*P* value	1.00	0.50	0.95	0.80	0.80	1.00	0.95

The data are presented as the means ± SDs.

### 3.5 Interaction analysis

**FIGURE 4:**
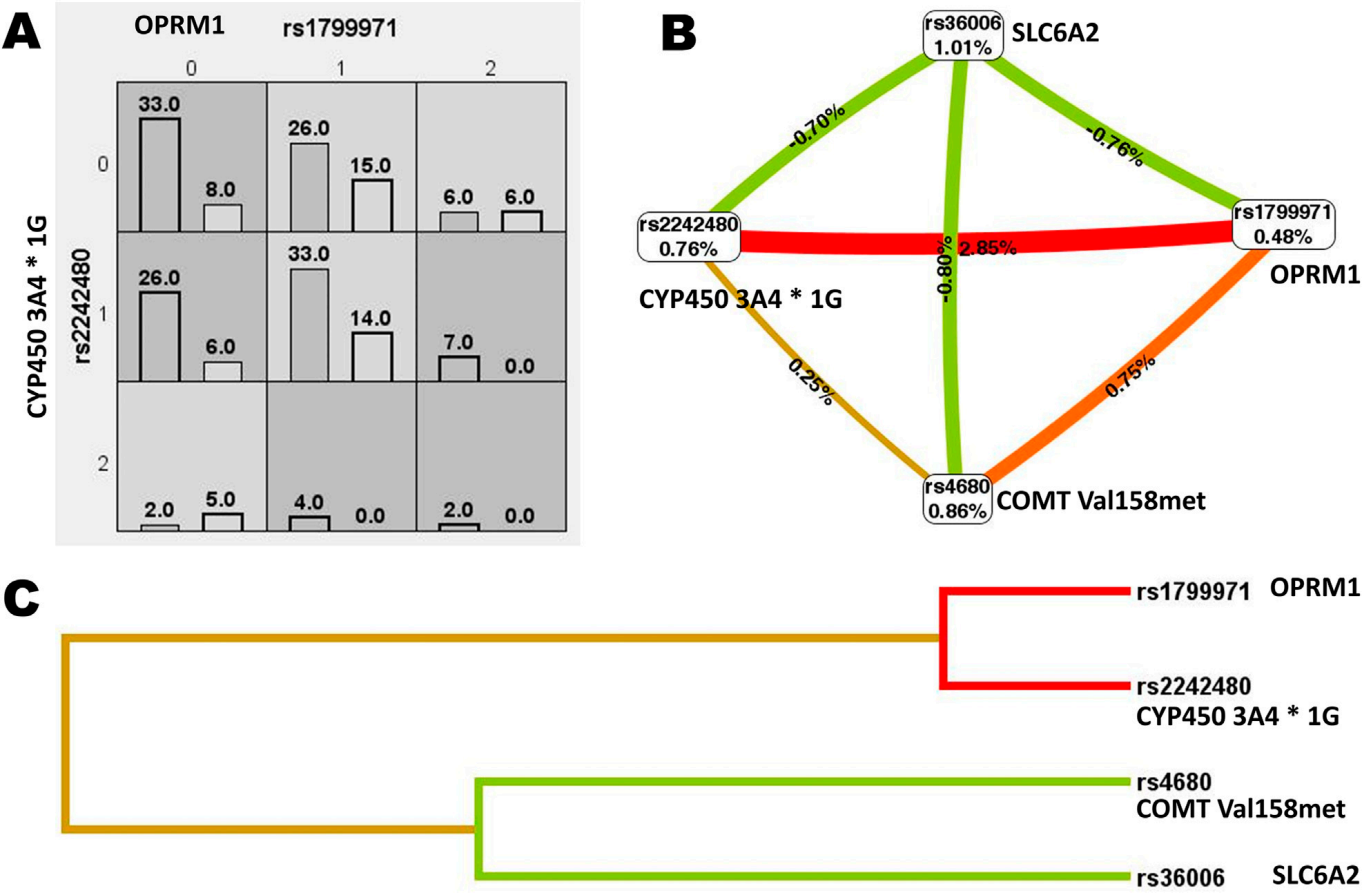
Gene interaction analysis. **(A)** Cell diagram showing the gene interaction analysis of *OPRM1 rs1799971* and CYP450 3A4*1G rs2242480. **(B)** Fruchterman-Reingold circular diagram illustrating the gene interaction analysis. The “n%” value between different SNP sites represents the strength of the interaction between two factors. A higher “n%” indicates a stronger interaction. **(C)** Tree diagram depicting the gene interaction analysis. Strong interactions were observed between two adjacent SNP sites, with the interaction weakening as the distance between them increased.

MDR was utilized to analyze the interactions among the *OPRM1 rs1799971*, CYP450 3A4 * 1G rs2242480, *SLC6A2 rs36006*, *SLC632 rs40615*, *COMT Val158met rs4680*, and *ADRA2A rs1800544*. The results revealed a significant interaction between the CYP450 3A4*1G (rs2242480) and *OPRM A118G (rs1799971)* loci (Figure 4). The MDR analysis identified that the optimal interaction model includes *rs179971* and *rs2242480* together. The accuracy of the training sample is 0.6301, and the accuracy of the validation sample is 0.6011. The cross-validation consistency was 10/10 (test χ^2^: 9.4778; p = 0.0021) (Table 9).

**TABLE 9:** Results of the analysis of high-order interactions of genetic polymorphic sites.

Model	Bal.Acc.CV training	Bal.Acc.CV testing	CV consistency	Test (*P*) value
*COMT Val158met.rs4680*	0.5656	0.4591	6/10	0.1188
CYP450 3A4*1G.rs2242480 and *OPRM A118G.rs1799971*	0.6301	0.6011	10/10	0.0021
CYP450 3A4*1G.rs2242480, *OPRM A118G.rs1799971* and *SLC6A2 rs36006*	0.6746	0.4123	5/10	0.0001

## 4 Discussion

This study investigated the correlation between several genetic polymorphisms and perioperative analgesia in Chinese patients undergoing gynecological surgery. The study found that the presence of the G mutation in *OPRM1 rs1799971* required higher doses of postoperative opioids. Patients with the *ADRA2A rs1800544* genotype carrying the G mutation had higher preoperative PCSs and required more remifentanil during surgery. These findings offer valuable information for tailoring opioid dosages for female patients undergoing gynecological procedures.

Previous studies have mainly focused on the *OPRM1 rs1799971* gene locus (Suresh et al., 2023). Research has shown that cancer patients with AA homozygosity of *OPRM1 A118G* require lower opioid doses for pain relief compared to those with G alleles (Yu et al., 2023). Turczynowicz A et al. (Turczynowicz et al., 2023) found that to achieve a similar analgesic effect, the dosage of sufentanil in the AG and GG groups was higher than in the AA group at two time points after surgery. Elgendy et al. (2023) reported that individuals carrying the G allele in OPRM1 rs1799971 exhibited higher postoperative pain scores. A meta-analysis found a strong association between the A118G mutation and cancer-related pain in Asian populations, but not in Caucasians (Hastie et al., 2012). This difference may be due to the higher prevalence of G allele carriers in Asian populations (40%–50%) compared to Caucasian populations (15%–30%) (Uhl et al., 1999). In our study, the frequency of the G allele among patients was 35%. Individuals with the G allele consumed more sufentanil than AA homozygous individuals, which is consistent with previous findings. However, the *OPRM1 rs1799971* gene polymorphism did not significantly correlate with preoperative pain sensitivity score, postoperative VAS score, Ramsay score, or side effects. Our results suggest no significant variation in PCS and PSQ scores among different genotypes of the OPRM1 gene.

Patients with the GG and AG genotypes of *OPRM1 rs1799971 (A118G)* typically require more postoperative analgesic drugs than those with the AA genotype due to several possible biological mechanisms. Firstly, the A118G polymorphism causes a specific alteration in the OPRM1 protein, replacing asparagine with aspartic acid at position 40. This change reduces the number of N-glycosylation sites on the μ-opioid receptor from 5 to 4 (Xie et al., 2022). N-glycosylation is essential for protein folding, stability, localization, and transport (Chen et al., 2023). This amino acid substitution may alter the overall conformation of the receptor, affecting the binding affinity of opioid drugs. The change in receptor conformation may reduce the coupling efficiency of the receptor with G proteins, weakening the receptor’s efficiency in activating downstream signaling pathways (Zhao et al., 2024). This reduction in functional activity means that the same dose of opioid drugs produces a weaker analgesic effect, necessitating higher doses for pain relief. Secondly, while there is no direct evidence that OPRM1 gene polymorphisms affect drug-metabolizing enzymes, studies suggest that genetic polymorphisms may indirectly alter opioid drug metabolism by influencing enzyme expression or activity (Kaneko et al., 2022). OPRM1 gene polymorphisms may also impact drug transporters, affecting the distribution and transport of opioid drugs in the body (Hwang et al., 2014). Inhibited drug transporter function reduces the efficiency of opioid drugs reaching their target sites in the central nervous system, requiring higher doses for effective analgesia. Furthermore, the *rs1799971* polymorphism may alter the binding efficiency of endogenous opioid peptides to MOR or the receptor’s sensitivity, affecting the endogenous opioid peptide system’s ability to inhibit pain signals (Walter and Lotsch, 2009). Patients with the G allele may have a reduced ability of the endogenous opioid peptide system to regulate pain, leading to lower pain tolerance post-surgery and a greater need for exogenous opioid drugs for analgesia supplementation.

The response to dexmedetomidine in adults has been linked to the *ADRA2A rs1800544* variant, with individuals carrying CC genotype showing a stronger sedative response compared to those with the GC and GG genotypes (Yagar et al., 2011). However, our study did not observe a correlation between ADRA2A polymorphisms and dexmedetomidine response. This lack of association may be attributed to the lower dosage of dexmedetomidine administered in our postoperative treatment compared to previous studies.

Patients with *ADRA2A rs1800544* CG and GG genotypes showed higher PCS scores than those with the CC genotype in this study. The PCS is a useful tool for assessing pain-related catastrophic thinking in both clinical practice and research (Perez-Cruzado et al., 2022). A higher PCS score suggests a greater requirement for analgesic treatment (Hayashi et al., 2022), which may explain the increased consumption of remifentanil among patients with the GG and CG genotypes. Additionally, there was a decreasing trend in postoperative hypotension in these patients compared to those with the CC genotype.

This study found no significant differences in adverse reactions between the mutation and wild-type groups for most SNPs. This lack of significance may be attributed to gene compensation mechanisms, insufficient sample size, or sample bias. The intricate gene regulatory networks in biological systems can obscure the effects of single nucleotide polymorphisms (SNPs) through compensatory mechanisms. When an SNP mutation occurs, other genes or pathways may compensate to maintain normal physiological functions, thereby masking the impact of the mutation (Vollger et al., 2025). Although the OPRM1 A118G polymorphism is not significantly associated with the incidence of adverse reactions such as nausea and vomiting, it is important to note that opioid dose is correlated with the occurrence of these side effects. Patients with the mutation may require higher doses of analgesics based on their genotype. In such instances, close monitoring for adverse reactions and adjusting antiemetic medication doses as necessary are crucial to optimize the pain management plan.

The interaction of SNP sites was analyzed using MDR. The results showed a strong interaction between the *OPRM1 rs1799971* and CYP450 3A4*1G rs2242480 sites, with the optimal interaction models including both *rs2242480* and *rs1799971*. This indicates that the combined effect of *OPRM1 rs1799971* and CYP450 3A4*1G rs2242480 variations has a more significant impact on postoperative VAS than their individual effects alone. Individuals carrying *rs1799971* and *rs2242480* at both SNP loci may have an increased risk for postoperative pain and may require personalized analgesia treatment.

This study has several limitations. Firstly, relying on patient self-rating scales for evaluating preoperative pain sensitivity may introduce subjectivity. Using advanced pain sensitivity indices, such as the high-frequency heart rate variability index (HFVi), could provide a more objective assessment. Secondly, the diversity in surgical procedures, patient demographics, outcome measures, and limited sample sizes have impacted the breadth and generalizability of our findings. Thirdly, the MassARRAY SNP genotyping technology may have technical limitations or assumptions that could potentially impact the research results. If the sample selection is not representative, it may lead to biased findings. Additionally, we did not investigate the relationship between single nucleotide polymorphisms (SNPs) and anesthetic analgesic effects at varying analgesic doses, representing an avenue for future research.

## 5 Conclusion

This study indicates that female patients undergoing gynecological surgery, especially those with the GG or AG genotype of the *OPRM1 rs1799971* gene, may need more 24-hour postoperative analgesia compared to those with the AA genotype. The identification of the OPRM1 A118G polymorphism holds promise for predicting personalized opioid dosages in gynecological surgery patients.

## Data Availability

The original contributions presented in the study are publicly available. This data can be found here: https://doi.org/10.5281/zenodo.15188935.
